# Positive association of triglyceride glucose index and gestational diabetes mellitus: a retrospective cohort study

**DOI:** 10.3389/fendo.2024.1475212

**Published:** 2025-01-06

**Authors:** Jie Zhang, Xia Fang, Zhan Song, Xue-ke Guo, Dong-mei Lin, Fei-na Jiang, Lin Lin, Zhu-hua Cai

**Affiliations:** Department of Obstetrics and Gynecology, The Third Affiliated Hospital of Wenzhou Medical University, Wenzhou, Zhejiang, China

**Keywords:** gestational diabetes mellitus, triglyceride glucose index, risk factor, prediction, metabolic biochemical markers

## Abstract

**Background:**

Gestational diabetes mellitus (GDM) is a common metabolic disorder with important health implications for both mother and offspring. This study aims to assess the relationship between the Triglyceride Glucose (TyG) index and GDM and explore its clinical significance.

**Methods:**

A retrospective cohort study included 631 singleton pregnant women. The study collected data on the TyG index, glucose levels, and clinical outcomes from all participants. Research objectives were validated using logistic regression analysis and Receiver Operating Characteristic curves.

**Results:**

We found an independent correlation between TyG index and increased risk of GDM, with an odds ratio (OR) of 3.11 (95% confidence interval (CI): 2.09-4.63, P<0.001). The spline model revealed a linear association between TyG index and GDM in early pregnancy (non-linear P=0.748), and the risk of GDM increased with the increase of TyG index. In addition, we found that TyG has high diagnostic performance in predicting GDM, with an AUC value of 0.668. Combined with Age, it can improve predictive performance, with an AUC value of 0.684. Compared to the lower quartile of the TyG index, women in the higher quartile have a higher incidence of premature birth, hypertensive disorders of pregnancy, and intrahepatic cholestasis of pregnancy (ICP). In the GDM group, the TyG index was negatively correlated with 25 (OH) D and TBIL, and positively correlated with FBG, TG, and TC.

**Conclusions:**

The TyG index is strongly linked to the development of GDM and is an independent risk factor for predicting it. Monitoring TyG levels in early pregnancy is valuable for identifying women at high risk for GDM.

## 1 Introduction

Gestational diabetes mellitus (GDM) is a form of glucose intolerance that develops or is first identified during pregnancy (1). It is among the most common metabolic complications experienced during pregnancy. This condition primarily affects the body’s carbohydrate metabolism but also involves changes in the metabolism of fats, proteins, and electrolytes. Gestational diabetes affects 9.3% - 25.5% of pregnant women depending on the characteristics of the population and the screening method applied (1, 2). The impact of GDM on pregnancy is influenced by the management of blood sugar levels. Inadequate blood sugar control can lead to complications related to blood vessels and infections, as well as premature birth, birth defects, and fetal death (3–5). Therefore, maintaining normal blood sugar levels is crucial for preventing these complications. The management of GDM typically involves strict dietary control and, in some cases, learning to self-monitor blood sugar levels and administer insulin. Maintaining appropriate blood glucose levels before and during pregnancy can significantly improve pregnancy outcomes and reduce the substantial risk of adverse outcomes for newborns. Presently, the diagnostic criteria for GDM primarily rely on oral glucose tolerance tests, which have limitations in terms of convenience, reproducibility, and predictive value for long-term metabolic complications (6).

Gestational diabetes mellitus (GDM) is a prevalent metabolic disorder during pregnancy, characterized by insulin resistance and relative insulin deficiency (2, 7). The presence of insulin resistance in patients with gestational diabetes mellitus (GDM) leads to an increase in maternal insulin demand. However, the islet beta cells are unable to effectively cope with this increased demand, resulting in elevated blood glucose levels (7). In normal pregnancy, beta cell proliferation and insulin secretion are physiologically increased to accommodate exacerbated insulin resistance (8). However, this dual defect of insulin resistance and insufficient islet beta cell function is central to the pathogenesis of GDM (9). Recent studies have suggested the Triglyceride Glucose (TyG) index as a potential surrogate marker for insulin resistance (10). The TyG index is calculated using fasting triglyceride and fasting glucose levels, which are physiologically altered during pregnancy to support fetal growth and development. The index reflects the body’s metabolic flexibility and ability to adapt to the changing energy demands of pregnancy.

Currently, more research is focused on women in mid pregnancy, such as Sánchez-García A (11), Salvatori B (12), Our study focuses on women who are 5-8 weeks pregnant, which is different from other literature. Son et al. found that the TyG index showed a higher predictive power than HOMA-IR in predicting metabolic syndrome (13). Yin et al. explored the efficacy of the TyG index and OGTT values in predicting the risk of gestational diabetes. They found that the TyG index may have similar or even higher efficacy than OGTT in predicting GDM (14). The HOMA-IR index is a classic index for assessing insulin resistance. However, insulin levels need to be measured; while OGTT is the gold standard for diagnosing GDM, the operation is complicated and has certain risks for subjects. The advantage of TyG index is that it only uses conventional blood lipid and blood glucose indicators, which is simpler and more economical. An elevated TyG index during early pregnancy has been found to predict an increased risk of GDM in the second trimester (11). The TyG index shows promise as an indicator for predicting diabetes (15, 16). However, the association between the TyG index and GDM has not been thoroughly investigated.

In our retrospective study, we analyzed data from 631 singleton pregnancies at the Third Affiliated Hospital of Wenzhou Medical University. Our focus was on examining the relationship between the 5-8 weeks gestation TyG index and the incidence of GDM. We conducted measurements of fasting metabolic biomarkers and employed rigorous statistical methods to evaluate the predictive capability of the TyG index for GDM.

## 2 Material and methods

### 2.1 Study participants

**Figure 1:**
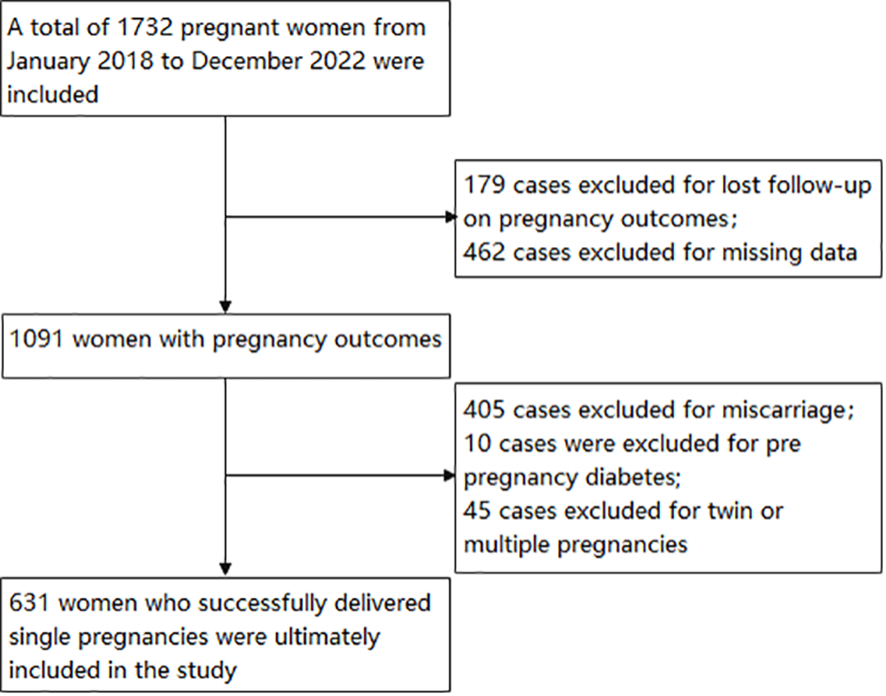
Flowchart of study participants. The figure showed the inclusion of participants.

Using a retrospective cohort study, 1732 pregnant women who visited the Third Affiliated Hospital of Wenzhou Medical University between 2018 and 2022 were included in the study. The inclusion criteria are pregnant women at 5-8 weeks of gestation. The exclusion criteria are: (1) Twin or multiple pregnancies; (2) The pregnancy outcome is lost to follow-up or the pregnancy outcome is abortion; (3) family history of diabetes mellitus; (4) diabetes mellitus occurring before pregnancy; (5) chronic diseases such as hypertension, cardiovascular disease, liver disease, kidney disease; (6) severe reproductive system infections; (7) Pregnant women who used drugs known to affect glucose or lipid metabolism during the study period; (8) cases with missing clinical data. Excluded from the study were cases of pregnancy outcomes lost to follow-up (n=179), miscarriage (n=405), twin or multiple pregnancies (n=45), prior diabetes mellitus (n=10), and missing data (n=462). The final analysis involved 631 pregnant women. **Figure 1** provides a visual representation of the study design and the flow of participants.

The research ethics of this study were approved by the ethics committee of The Third Affiliated Hospital of Wenzhou Medical University (No. YJ2024106).

### 2.2 Measurements and definitions

Serum samples from all participants were collected on admission. After a fasting period of over 8 hours, the height, weight and blood pressure were measured the following morning. A 10ml sample of peripheral venous blood was collected to determine various metabolic-related biochemical indicators and inflammatory markers. Using a full-automatic biochemical analyzer (Siemens CH930) to detect fasting blood glucose (FBG), total cholesterol (TC), triglyceride (TG), high density lipoprotein (HDL), low density lipoprotein (LDL), total bilirubin (TBIL), alanine transaminase (ALT), aspartate transaminase (AST), urea, serum creatinine (SCR), uric acid (UA), and homocysteine (HCY). Additionally, the TyG index was calculated using the formula ln (fasting TG [mg/dL] * fasting glucose [mg/dL]/2) (17). The concentration of 25 Hydroxyvitamin D [25(OH)D] in the serum of patients was measured using the liquid chromatography-tandem mass spectrometry method (Siemens IM1600, Shanghai, China). The levels of plasma cytokines Interleukin 6 (IL-6) and tumor necrosis factor-alpha (TNF-a) were detected by flow cytometry (BD FACSCanto II, Shanghai, China). Use Sysmex XN-1000 blood cell analyzer to detect White blood cell (WBC), neutrophil counts, and lymphocyte counts. Neutrophil-to-lymphocyte ratio (NLR), calculated as the ratio between neutrophil and lymphocyte counts measured in peripheral blood.

### 2.3 Diagnosis of incident GDM

An oral glucose tolerance test should be conducted following the Chinese GDM diagnostic guidelines (2022) (18). The test should be performed between 24-28 weeks of pregnancy. Prior to the test, it is important to maintain a normal diet for 3 consecutive days, ensuring a daily intake of no less than 150 g of carbohydrates. Fasting for 8-10 hours before the test is necessary, and it is important to refrain from sitting and smoking during the examination. During the test, 300 ml of liquid containing 75 g of glucose (anhydrous glucose powder) should be orally ingested within 5 minutes. Venous blood samples should be taken before and 1 hour and 2 hours after glucose administration (calculated from the start of glucose water consumption) and placed in a test tube containing sodium fluoride. The plasma glucose level should be measured using the glucose oxidase method. A diagnosis of GDM is made if any of the following criteria are met or exceeded: fasting blood glucose level 5.1 mmol/L (91.90 mg/dL), 1-hour blood glucose level 10.0 mmol/L (180.20 mg/dL), or 2-hour blood glucose level 8.5 mmol/L (153.17 mg/dL). These standards align with the latest guidelines of the International Association of Diabetes and Pregnancy Research Groups (19).

### 2.4 Statistical analysis

All statistical analyses were conducted using R version 3.3.2 (https://www.r-project.org/, The R Foundation) and Free Statistics software version 1.9.2. Two-tailed tests were performed with a significance level of p < 0.05. The distribution of TyG values was divided into four groups, from the lowest quartile (Q1) to the highest quartile (Q4). Participant data was analyzed based on these quartiles. Normality was assessed using the Shapiro-Wilker test and histogram shape. Normally distributed continuous variables were presented as mean ± standard deviation, while skewed continuous variables were reported as median (interquartile range [IQR]). Categorical variables were expressed as frequencies and percentages (n (%)). Differences between TyG index groups were evaluated using the Kruskal-Wallis test, chi-square test, and one-way ANOVA. A logistic regression analysis was conducted to assess whether the TyG index was an independent risk factor, with GDM (presence/absence) as the dependent variable and the TyG index as a continuous variable representing clinical diagnosis. Confounders were selected based on clinical relevance and significance in univariate analysis, and three models were constructed. Model 1 had no adjustments, and Model 2 included adjustments for Age, BMI, SBP, DBP, previous parity, and previous miscarriages, and Model 3 further included adjustments for TC, HDL, LDL, TBIL, and UA. Spline regression analysis was used to determine the continuous association of the TyG index and GDM incidence. Receiver operating characteristic (ROC) curves were used to assess the predictability of GDM.

## 3 Results

### 3.1 Characteristics of participants

In **Table 1**, the baseline characteristics were analyzed based on the quartiles of the TyG index. It was observed that Age, SBP, DBP, BMI, FBG, TG, LDL, ALT, UA, WBC and GDM rates increased as the quartiles of the TyG index increased (*P <*0.001). Conversely, TBIL decreased with higher TyG index quartiles. Women in the higher quartile exhibited higher previous parity, TC, NLR, IL-6 and TNF-α compared to those in the lower quartile of the TyG index (*P <*0.05). However, there were no significant differences in previous miscarriages, HCY, VD, HDL, AST, urea, and birth weight (all *P* values > 0.05).

**Table 1:** The baseline characteristics of participants.

Variables	Total (n = 631)	TyG index				*P*-value
		Q1 (n = 158)	Q2 (n = 157)	Q3 (n = 158)	Q4 (n = 158)	
Age (years)	28.00 (26.00, 32.00)	27.00 (25.00,30.00)	28.00 (26.00,30.00)	29.00 (26.00,31.75)	30.00 (27.00,33.00)	**<0.001**
SBP (mm/Hg)	116.1 ± 11.9	113.3 ± 11.5	115.4 ± 12.1	116.4 ± 10.4	119.3 ± 12.6	**<0.001**
DBP (mm/Hg)	70.00 (64.00, 77.00)	68.00 (62.00,74.75)	69.00 (64.00,74.00)	70.00 (65.00,77.00)	73.00 (67.00,79.00)	**<0.001**
BMI (kg/m^2^)	20.96 (19.23, 23.23)	20.05 (18.82,21.48)	20.39 (18.89,22.27)	21.36 (19.68,23.50)	22.67 (20.40,25.38)	**<0.001**
Previous parity						**0.008**
Nulliparous	427 (67.7)	113 (71.5)	117 (74.5)	106 (67.1)	91 (57.6)	
Parous	204 (32.3)	45 (28.5)	40 (25.5)	52 (32.9)	67 (42.4)	
Previous miscarriages						0.151
0	200 (31.7)	37 (23.4)	52 (33.1)	53 (33.5)	58 (36.7)	
1	215 (34.1)	67 (42.4)	50 (31.8)	50 (31.6)	48 (30.4)	
≥2	216 (34.2)	54 (34.2)	55 (35)	55 (34.8)	52 (32.9)	
HCY (umol/L)	6.00 (5.40, 6.80)	6.10 (5.50,6.90)	6.10 (5.50,6.70)	5.90 (5.30,7.00)	5.90 (5.30,6.70)	0.369
25(OH)D(ng/ml)	17.93 (13.50, 23.10)	19.62 (14.84,23.67)	17.40 (13.90,23.19)	17.40 (12.72,21.82)	17.95 (13.41,23.17)	0.13
FBG (mmol/L)	4.49 (4.08, 4.99)	4.22 (3.79,4.60)	4.50 (4.21,4.90)	4.53 (4.01,4.91)	4.71 (4.26,5.45)	**<0.001**
TC (mmol/L)	3.86 (3.41, 4.39)	3.42 (3.12,3.95)	3.88 (3.47,4.27)	3.81 (3.46,4.34)	4.31 (3.83,4.97)	**<0.001**
TG (mmol/L)	0.94 (0.70, 1.35)	0.60 (0.51,0.70)	0.82 (0.72,0.90)	1.13 (1.01,1.30)	1.73 (1.44,2.11)	**<0.001**
HDL (mmol/L)	1.27 (1.07, 1.46)	1.27 (1.08,1.44)	1.29 (1.10,1.49)	1.23 (1.01,1.43)	1.26 (1.08,1.50)	0.362
LDL (mmol/L)	2.15 (1.80, 2.58)	1.92 (1.61,2.24)	2.18 (1.84,2.57)	2.21 (1.85,2.60)	2.40 (1.94,3.04)	**<0.001**
TBIL (umol/L)	9.50 (7.10, 12.80)	11.05 (8.60,13.97)	10.80 (7.80,14.10)	9.35 (6.93,12.52)	7.40 (6.40,9.65)	**<0.001**
ALT(U/L)	14.00 (10.00, 22.00)	12.00 (9.00,17.75)	14.00 (10.00,19.00)	15.00 (10.00,23.75)	17.00 (11.00,31.00)	**<0.001**
AST(U/L)	16.00 (14.00, 20.00)	16.00 (14.00,18.75)	16.00 (13.00,19.00)	16.00 (14.00,21.00)	16.50 (13.00,22.00)	0.448
Urea(mmol/L)	3.54 (2.91, 4.29)	3.68 (3.16,4.29)	3.44 (2.98,4.08)	3.54 (2.81,4.31)	3.50 (2.71,4.53)	0.194
SCR (umol/L)	50.8 ± 8.2	50.5 ± 7.7	51.3 ± 8.0	50.3 ± 8.4	51.2 ± 8.7	0.667
UA (umol/L)	244.00 (204.00, 284.50)	237.00 (199.00,272.75)	238.00 (202.00,281.00)	239.00 (204.25,283.75)	259.00 (222.00,303.50)	**0.001**
WBC (10^9/L)	8.1 (6.5, 10.7)	7.2 (6.0, 9.9)	8.0 (6.2, 10.4)	8.4 (6.7, 10.6)	8.6 (7.2, 11.2)	**<0.001**
NLR	2.8 (2.1, 4.1)	2.7 (1.9, 4.2)	2.9 (2.0, 4.3)	2.8 (2.1, 3.9)	2.9 (2.2, 4.1)	0.673
IL-6 (pg/ml)	2.4 (1.5, 3.7)	2.0 (1.4, 3.3)	2.7 (1.7, 3.7)	2.3 (1.5, 3.5)	2.9 (1.8, 4.4)	**0.006**
TNF-α(pg/ml)	0.8 (0.5, 2.0)	0.7 (0.4, 1.9)	0.9 (0.5, 3.1)	0.7 (0.4, 1.4)	0.9 (0.5, 2.2)	**0.023**
Birth weight(g)	3113.4 ± 529.4	3089.8 ± 497.0	3146.3 ± 510.2	3136.7 ± 526.8	3081.0 ± 581.6	0.612
GDM						**<0.001**
NO	529 (83.8)	147 (93)	138 (87.9)	131 (82.9)	113 (71.5)	
YES	102 (16.2)	11 (7)	19 (12.1)	27 (17.1)	45 (28.5)	

Data presented are mean ± SD, median (IQR), or n (%).

TyG, Triglyceride Glucose; SBP, Systolic blood pressure; DBP, Diastolic blood pressure; BMI, Body mass index; HCY, Homocysteine; 25(OH)D, 25 Hydroxyvitamin D; FBG, fasting blood glucose; TC, total cholesterol; TG, triglyceride; HDL, high density lipoprotein; LDL, low density lipoprotein; TBIL, total bilirubin; ALT, alanine transaminase; AST, aspartate transaminase; SCR, serum creatinine; UA, uric acid; WBC, White blood cell; NLR, neutrophil-to-lymphocyte ratio; IL-6, Interleukin 6; TNF-α, tumor necrosis factor-alpha; GDM, Gestational diabetes mellitus.

Bold values are statistical significance (p < 0.05).

### 3.2 The results of univariate analyses

The findings from the univariate analysis are detailed in **Table 2**. The analysis revealed positive correlations between GDM occurrence and Age, DBP, BMI, Previous miscarriages, FBG, CHO, TG, LDL, and TyG index. Additionally, there was a negative correlation observed between TBIL and the incidence of GDM.

**Table 2:** The results of the univariate analysis.

Variable	OR_95CI	P-value
Age(years)	1.09 (1.04~1.14)	**<0.001**
SBP (mm/Hg)	1.02 (1~1.04)	**0.013**
DBP (mm/Hg)	1.04 (1.02~1.06)	**0.001**
BMI (kg/m^2^)	1.16 (1.08~1.23)	**<0.001**
Previous parity	1.13 (0.77~1.66)	0.531
Previous miscarriages	1.2 (1.03~1.4)	**0.02**
HCY (umol/L)	0.9 (0.76~1.06)	0.205
25(OH)D(ng/ml)	1.01 (0.98~1.04)	0.718
FBG (mmol/L)	1.84 (1.48~2.28)	**<0.001**
TC (mmol/L)	1.55 (1.22~1.96)	**<0.001**
TG (mmol/L)	1.49 (1.16~1.91)	**0.002**
HDL (mmol/L)	0.95 (0.47~1.9)	0.876
LDL (mmol/L)	1.72 (1.29~2.28)	**<0.001**
TBIL (umol/L)	0.92 (0.87~0.97)	**0.002**
ALT(U/L)	1 (1~1.01)	0.232
AST(U/L)	1.01 (1~1.02)	0.172
Urea(mmol/L)	1.15 (0.97~1.37)	0.114
SCR (umol/L)	1 (0.97~1.02)	0.733
UA (umol/L)	1 (1~1.01)	**0.005**
WBC (10^9/L)	1.01(0.97~1.05)	0.609
NLR	1.02(0.95~1.1)	0.581
IL-6 (pg/ml)	0.99(0.96~1.02)	0.501
TNF-α(pg/ml)	1(0.99~1.02)	0.508
TyG index	3.11 (2.09~4.63)	**<0.001**

SBP, Systolic blood pressure; DBP, Diastolic blood pressure; BMI, Body mass index; HCY, Homocysteine; 25(OH)D, 25 Hydroxyvitamin D; FBG, fasting blood glucose; TC, total cholesterol; TG, triglyceride; HDL, high density lipoprotein; LDL, low density lipoprotein; TBIL, total bilirubin; ALT, alanine transaminase; AST, aspartate transaminase; SCR, serum creatinine; UA, uric acid; WBC, White blood cell; NLR, neutrophil-to-lymphocyte ratio; IL-6, Interleukin 6; TNF-α, tumor necrosis factor-alpha; TyG, Triglyceride Glucose.

Bold values are statistical significance (p < 0.05).

### 3.3 TyG index and GDM risk

The study utilized multiple logistic regression analysis to investigate the relationship between the TyG index and GDM (as shown in **Table 3**). In Model 1, a positive correlation was found between the TyG index and the occurrence of GDM (OR: 3.11, 95% CI: 2.09 – 4.63, *P* < 0.001). Model 2, which was adjusted for Age, BMI, SBP, DBP, previous parity, and previous miscarriages, revealed that each 1 unit increase in the TyG index was associated with a 2.3-fold increase in GDM risk. The highest quartile exhibited the most significant GDM risk compared to the lowest quartile (OR: 3.5; 95% CI: 1.65 to 7.43). This association became more pronounced in model 3, which further adjusted for TC, HDL, LDL, TBIL, and UA. Across all models, there was a consistent pattern of progressively increasing GDM risk with each quartile of the TyG index (*P* < 0.05).

**Table 3:** Association between TyG index and GDM.

	Model 1		Model 2		Model 3	
	OR (95%CI)	*P*-value	OR (95%CI)	*P-*value	OR (95%CI)	*P*-value
TyG index	3.11 (2.09~4.63)	<0.001	2.3 (1.48~3.59)	<0.001	2.26 (1.27~4.02)	0.005
TyG index, Quartile						
Quartile 1	1(Ref)		1(Ref)		1(Ref)	
Quartile 2	1.84 (0.85~4.01)	0.125	1.7 (0.77~3.74)	0.188	1.68 (0.75~3.75)	0.205
Quartile 3	2.75 (1.31~5.77)	0.007	2.17 (1.01~4.63)	0.047	1.94 (0.89~4.24)	0.094
Quartile 4	5.32 (2.63~10.75)	<0.001	3.5 (1.65~7.43)	0.001	2.67 (1.17~6.07)	0.019
Trend.test	1.73 (1.4~2.13)	<0.001	1.49 (1.19~1.87)	0.001	1.35 (1.05~1.73)	0.02

Model 1: Unadjusted.

Model 2: Adjusted for Age、BMI、SBP、DBP、previous parity and previous miscarriages.

Model 3: Further adjusted for TC、HDL、LDL、TBIL、UA.

TyG, Triglyceride Glucose; GDM, Gestational diabetes mellitus; BMI, Body mass index; SBP, Systolic blood pressure; DBP, Diastolic blood pressure; TC, total cholesterol; HDL, high density lipoprotein; LDL, low density lipoprotein; TBIL, total bilirubin; UA, uric acid.

### 3.4 The results of the subgroup analysis

**Figure 2:**
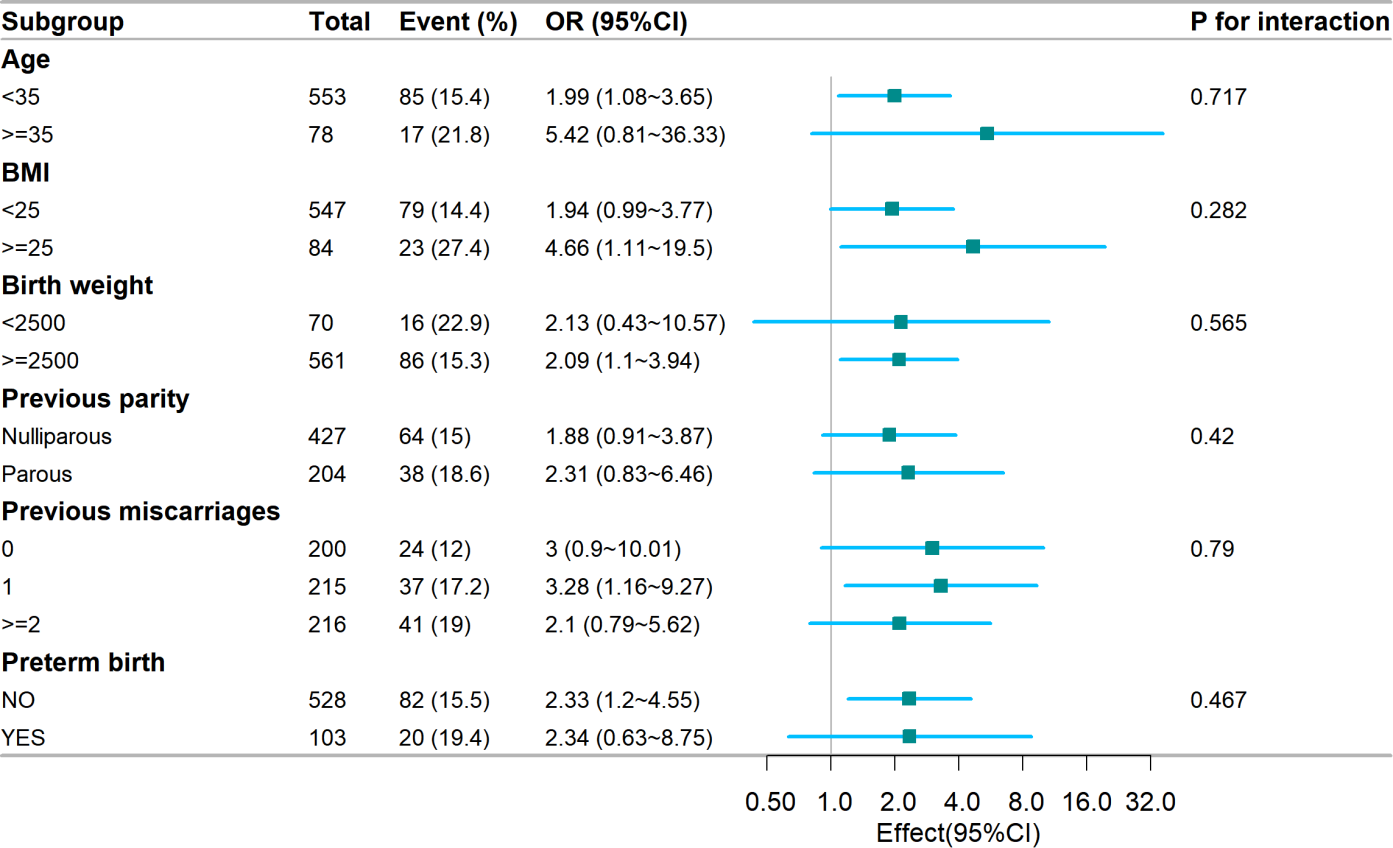
Subgroup analysis of the association of the TyG index and GDM risk. Models were adjusted according to Age, BMI, blood pressure, miscarriage, delivery, TC, uric acid, LDL, total bilirubin, and HDL. TyG, Triglyceride Glucose; GDM, Gestational diabetes mellitus; BMI, Body mass index; TC, total cholesterol; LDL, low density lipoprotein; HDL, high density lipoprotein.

We conducted subgroup analysis based on various factors to gain further insight into the relationship (refer to **Figure 2**) between the TyG index and GDM risk, with the aim of identifying potential confounding variables that could impact the findings. Age, BMI, birth weight, previous parity, previous miscarriages, and premature birth were chosen as the stratification factors. Our analysis revealed that these potential confounders did not alter the association between the TyG index and GDM risk. The results of the subgroup analysis affirm the strength of our conclusions.

### 3.5 The TyG index in early pregnancy is closely associated with the incidence of GDM

**Figure 3:**
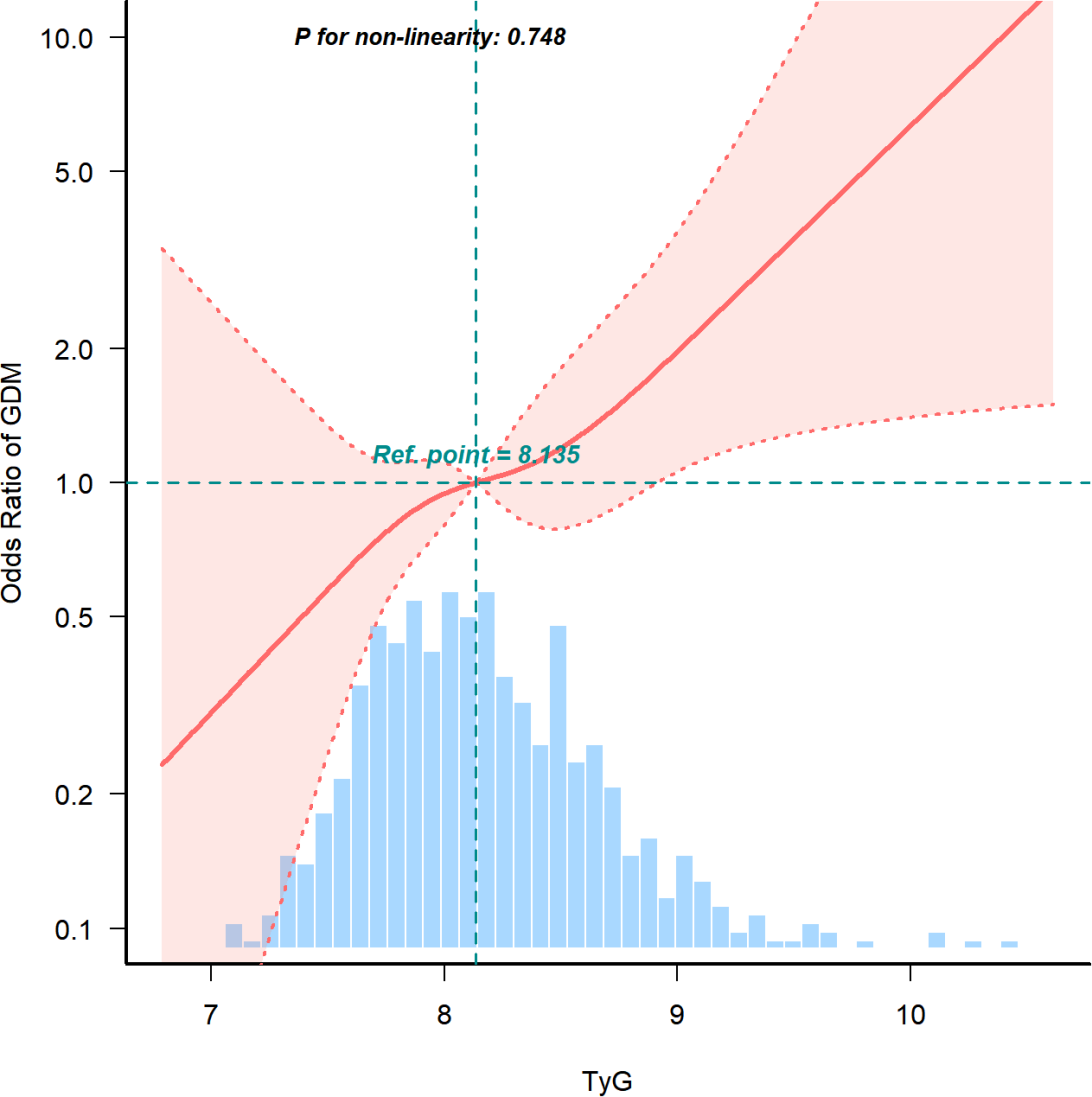
Continuous association of the TyG index with the incidence of GDM. Adjusted for Age, BMI, blood pressure, miscarriage, delivery, TC, uric acid, LDL, total bilirubin, and HDL. TyG, Triglyceride Glucose; GDM, Gestational diabetes mellitus; BMI, Body mass index; TC, total cholesterol; LDL, low density lipoprotein; HDL, high density lipoprotein.

After adjusting for age, BMI, blood pressure, history of miscarriage, delivery, TC, UA, LDL, TBIL, and HDL, the spline model revealed a linear association between the TyG index and GDM in early pregnancy (**Figure 3**, P for non-linearity = 0.748). The risk of GDM increased as the TyG index levels increased.

### 3.6 ROC curve analysis of the TyG index, early pregnancy BMI, FBG, and TG for predicting GDM

**Figure 4:**
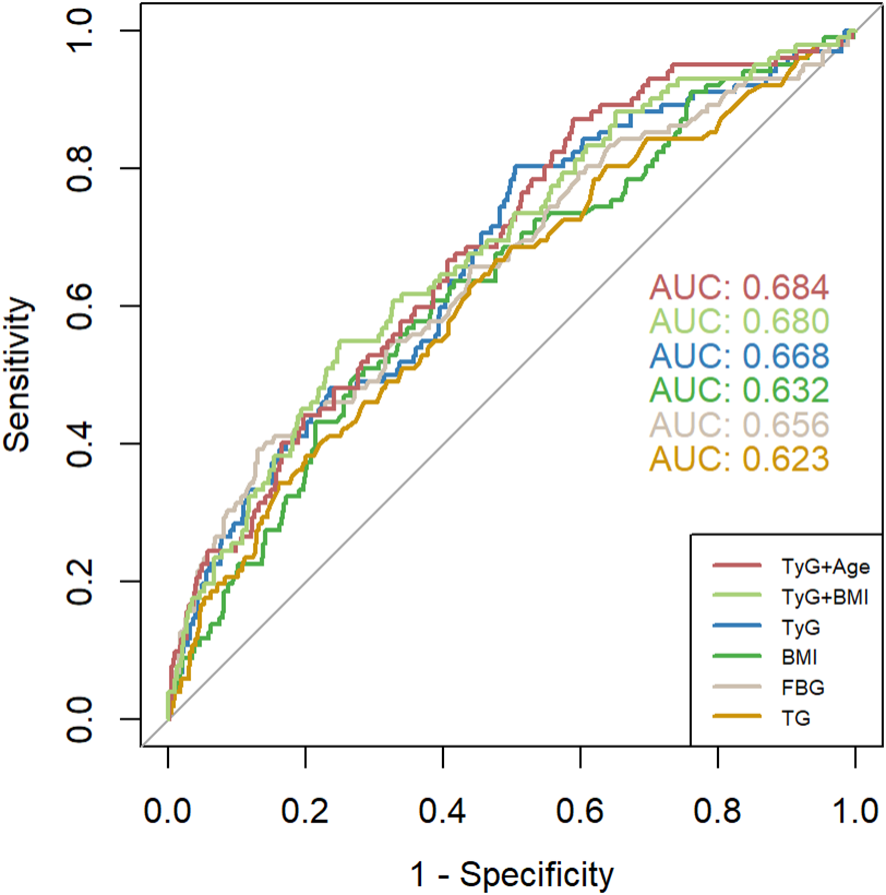
ROC curves of TyG index, BMI 、FBG, and TG for predicting the development of GDM. TyG, Triglyceride Glucose; TG, triglyceride; BMI, Body mass index; FBG, fasting blood glucose.

ROC analysis was performed to predict GDM, and the results showed that the AUC of TyG was 0.668 (95% CI: 0.610-0.726), the AUC of BMI was 0.632 (95% CI: 0.572-0.692), the AUC of FBG was 0.656 (95% CI: 0.594-0.717), and the AUC of TG was 0.623 (95% CI: 0.562-0.684). The TyG index showed the highest AUC predicting GDM compared to other factors such as TG, FBG, and BMI. Using the Jorden index, the optimal cutoff point for the TyG index to predict GDM was determined to be 8.066. At this threshold, the specificity was 49.5% and the sensitivity was 80.4%. When using joint indicators, the AUC of TyG + Age is the highest, at 0.684 (95% CI: 0.628~0.739), and the AUC of TyG + BMI is 0.680 (95% CI: 0.623~0.738) (**Figure 4**).

### 3.7 The association between TyG index and other adverse pregnancy outcomes

In terms of adverse pregnancy outcomes, the TyG index in the hypertensive disorders of pregnancy group was (8.4 ± 0.5), significantly higher than that in the non-hypertensive disorders of pregnancy group (P<0.05). There was no significant difference in TyG index outcomes for preterm birth, macrosomia, ICP, and premature rupture of membranes. Women in the higher quartile exhibited higher rates of preterm birth, Hypertensive disorders of pregnancy and ICP compared to those in the lower quartile of the TyG index (P <0.05), while it shows a lower rate of premature rupture of membranes (**Table 4**).

**Table 4:** Association between TyG index and other adverse pregnancy outcomes.

Characteristic	TyG index	*P*-value	TyG index				*P*-value
			Q1 (n = 158)	Q2 (n = 157)	Q3 (n = 158)	Q4 (n = 158)	
preterm birth		0.278					**0.043**
NO	8.2 ± 0.5		128 (81)	135 (86)	141 (89.2)	124 (78.5)	
YES	8.2 ± 0.6		30 (19)	22 (14)	17 (10.8)	34 (21.5)	
macrosomia		0.203					0.509
NO	8.2 ± 0.5		154 (97.5)	150 (95.5)	154 (97.5)	150 (94.9)	
YES	8.3 ± 0.5		4 (2.5)	7 (4.5)	4 (2.5)	8 (5.1)	
Hypertensive disorders of pregnancy		**0.002**					**0.027**
NO	8.2 ± 0.5		150 (94.9)	150 (95.5)	149 (94.3)	139 (88)	
YES	8.4 ± 0.5		8 (5.1)	7 (4.5)	9 (5.7)	19 (12)	
ICP		0.059					**0.026**
NO	8.2 ± 0.5		155 (98.1)	156 (99.4)	158 (100)	152 (96.2)	
YES	8.5 ± 0.8		3 (1.9)	1 (0.6)	0 (0)	6 (3.8)	
Premature rupture of membranes		0.194					**0.015**
NO	8.2 ± 0.5		132 (83.5)	119 (75.8)	133 (84.2)	141 (89.2)	
YES	8.1 ± 0.6		26 (16.5)	38 (24.2)	25 (15.8)	17 (10.8)	

TyG, Triglyceride Glucose; ICP, Intrahepatic cholestasis of pregnancy.

Bold values are statistical significance (p < 0.05).

### 3.8 The correlation between TyG index and metabolic indicators and inflammatory markers in all pregnant women and GDM groups

**Figure 5:**
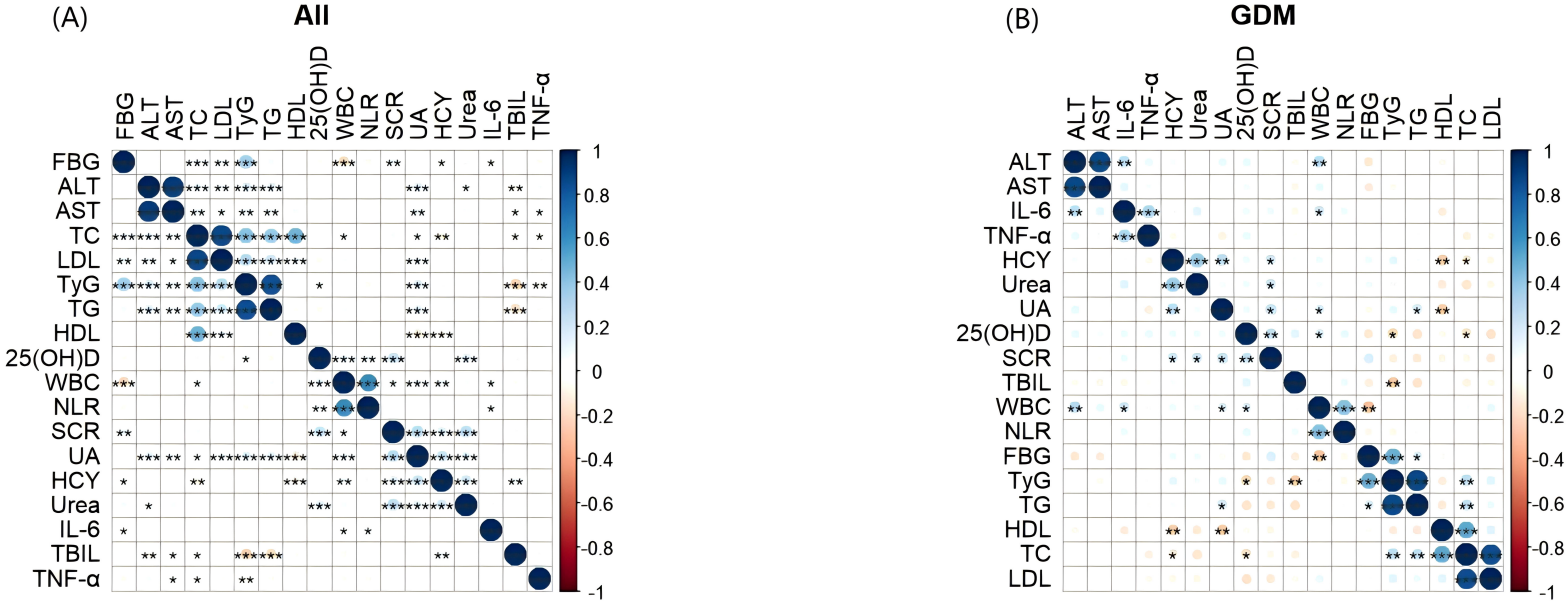
Heatmap of correlation and significance between TyG index and metabolic indicators, inflammatory markers. The two-color heat map visually represents the association between TyG index, and metabolic indicators, inflammatory markers with blue indicating positive correlation and red indicating negative correlation. Asterisks denote significance: *p < 0.05; **p < 0.01; ***p < 0.001. **(A)** Heatmap of correlation and significance between TyG index and metabolic indicators, inflammatory markers in all pregnant groups. **(B)** Heatmap of correlation and significance between TyG index and metabolic indicators, inflammatory markers in the GDM groups. TyG, Triglyceride Glucose; HCY, Homocysteine; 25(OH)D, 25HydroxyvitaminD; FBG, fasting blood glucose, TC: total cholesterol; TG, triglyceride; HDL, high density lipoprotein; LDL, low density lipoprotein; TBIL, total bilirubin; ALT, alanine transaminase; AST, aspartate transaminase; SCR, serum creatinine; UA, uric acid; WBC, White blood cell; NLR, neutrophil-to-lymphocyte ratio; IL-6, Interleukin 6; TNF-α, tumor necrosis factor-alpha.

In all pregnant groups, the TyG index was positively correlated with FBG, ALT, AST, TC, LDL, TG and UA, and negatively correlated with 25 (OH) D, TBIL and TNF-α. In the GDM group, the TyG index was negatively correlated with 25 (OH) D and TBIL, and positively correlated with FBG, TG and TC (**Figure 5**).

The two-color heat map visually represents the association between TyG index, and metabolic indicators, inflammatory markers with blue indicating positive correlation and red indicating negative correlation. Asterisks denote significance: *p < 0.05; **p < 0.01; ***p < 0.001. (A) Heatmap of correlation and significance between TyG index and metabolic indicators, inflammatory markers in all pregnant groups. (B) Heatmap of correlation and significance between TyG index and metabolic indicators, inflammatory markers in the GDM groups.

## 4 Discussion

GDM is a condition characterized by glucose intolerance that occurs or is first recognized during pregnancy (1, 2). It presents significant health risks for both the mother and the fetus, including an increased likelihood of cesarean delivery, preeclampsia, and the development of type 2 diabetes mellitus later in life for the mother. Additionally, it can lead to macrosomia, neonatal hypoglycemia, and an elevated risk of obesity and diabetes for the child (19, 20).With the global increase in GDM prevalence, it is essential to identify reliable biomarkers for early detection and effective management of this condition (21).

In recent years, numerous studies have demonstrated that the TyG index is linked to various health issues such as cardiovascular disease, diabetes, certain types of cancer, and cognitive decline (22). However, its correlation with GDM has not been thoroughly investigated. In our study, we have observed a significant positive association between the TyG index and the risk of GDM, consistent with results from other observational studies (23).Our results have shown consistency across various clinical subgroups. This suggests that TyG is an independent risk factor for GDM. Moreover, the high accuracy of the TyG index in predicting GDM, as demonstrated by ROC curve analysis, emphasizes its potential usefulness in clinical settings for risk assessment and targeted prevention strategies. Previous research has investigated the link between the TyG index and GDM. For example, Zihe Mo et al. (23) reported a significant association between the TyG index and an increased risk of GDM in the Korean population (OR = 12.923, 95%CI: 3.581–46.632). Additionally, a recent retrospective cohort study involving Korean primiparous women demonstrated a significant relationship between the TyG index and the occurrence of GDM (24). Furthermore, a recent prospective cohort study confirmed that the TyG index can effectively identify the occurrence of GDM in the second trimester (25). This analysis demonstrates the correlation between the TyG index and the incidence rate of GDM. The possible mechanisms by which TyG affects GDM are as follows (1):Dysregulated lipid metabolism can lead to the accumulation of lipotoxic mediators that enhance insulin signaling (26). And in beta cells, excessive free fatty acids (FFA) can cause lipotoxicity, which involves disturbances in intracellular lipid metabolism, leading to the production of reactive oxygen species (ROS) and endoplasmic reticulum (ER) stress, thereby interfering with insulin signaling and inhibiting insulin secretion (27) (2). Chronic low-grade inflammation, often linked to obesity, can worsen insulin resistance by releasing pro-inflammatory cytokines (28, 29). Oxidative stress, caused by an imbalance between reactive oxygen species production and antioxidant defenses, can also disrupt insulin action (30).

This study also analyzed TyG and other adverse perinatal outcomes and found that as the TyG index increased, the risk of preterm birth, gestational hypertension, ICP, and premature rupture of membranes increased. Zhang et al. (31) found a positive independent correlation between TyG index and preeclampsia, premature birth, and macrosomia. They proposed that insulin directly or indirectly promotes premature birth through inflammation, oxidative stress, and endothelial dysfunction. Overactivation of insulin resistance can stimulate the sympathetic nervous system, increase vascular resistance, increase sodium reabsorption in distal glomeruli, ultimately leading to endothelial dysfunction and potentially causing elevated blood pressure during pregnancy (32).

This study also revealed that TyG is negatively correlated with 25 (OH) D and TBIL, and positively correlated with FBG, TG, and TC. Studies have shown that low levels of vitamin D are associated with insulin resistance, which is the biological basis for elevated TyG index. Therefore, the TyG index may be negatively correlated with 25 (OH) D levels (33). Fasting blood glucose and triglycerides are components of the TyG index, so there is a positive correlation between them. Insulin resistance can affect lipid metabolism, including cholesterol metabolism, so the TyG index may be positively correlated with total cholesterol levels.

The study provides valuable insights into the relationship between the TyG index and the incidence of GDM. Furthermore, the study population in this research consists of women who are 5-8 weeks pregnant, which allows for the early identification of GDM risk, potentially contributing to timely intervention and improved pregnancy outcomes. However, it has several limitations that should be considered. Firstly, the study’s use of a retrospective cohort design may introduce recall bias. Secondly, the TyG index is only measured once in the early stages of pregnancy, potentially overlooking fluctuations throughout the entire pregnancy period. Thirdly, the focus on Ruian, a more developed economic region, may limit the generalizability of the results. In addition, the study lacks confounding controls for lifestyle factors, including dietary habits, exercise levels, and so on. Considering that diet and exercise are key lifestyle factors that affect insulin sensitivity and glucose metabolism. In our future research, we need to address these shortcomings through more comprehensive lifestyle assessments. This may include using food diaries, exercise trackers, or wearable devices to objectively measure diet and exercise. Although we do not have specific dietary and exercise data, we still attempt to control for potential confounding by adjusting known confounding factors such as BMI, blood pressure, and lipid levels. These variables may indirectly reflect the impact of lifestyle. Future research can further explore the predictive efficacy of TyG index in different races and populations, and consider combining it with other biomarkers to improve the accuracy and reliability of predictions. In addition, the dynamic changes of TyG index in early pregnancy and its relationship with GDM can also be studied, as well as how it affects the long-term health of pregnant women and newborns. Our research shows a significant correlation between TyG index and GDM, and TyG index can serve as a predictive indicator for GDM. GDM is a pregnancy specific disease that has potential long-term effects on the health of both pregnant women and fetuses. By using the TyG index, we can identify high-risk populations for GDM in early pregnancy, enabling timely intervention and reducing the risk of maternal and infant complications. In addition, the measurement of TyG index is relatively simple and cost-effective, making it suitable for use in resource limited environments. Future research should focus on verifying these results in larger and more diverse populations, and studying the potential mechanisms by which TyG index affects GDM risk, ultimately improving our ability to prevent and manage such situations.

## Data Availability

The original contributions presented in the study are included in the article. Further inquiries can be directed to the corresponding authors.
